# Use of Capsicum in Delirium Tremens

**Published:** 1869-04

**Authors:** R. D. Lyon


USE OF CAPSICUM IN DELIRIUM TREMENS.—By R. D. LYON, M. B., in *British Medical Journal*, November 7.

Dr. Lyon sums up his experience of the drug as follows: 1. Capsicum is a valuable and reliable drug when opium fails, or is, for any cause, contra-indicated. 2. It is a safe drug for general employment in delirium tremens, and as such, may be confidently recommended to the country practitioner for general employment. 3. It is not open to the objection which attaches to the continued use of opium, which, when it fails to tranquilize and produce sleep, adds to the state of excitement; and, if pursued beyond a certain limit, kills, as it has undoubtedly done in numerous instances, by suddenly induced opium-coma. 4. In some few instances, Dr. Lyon informs us, he has employed capsicum in the delirium of fever, when opium had failed to induce sleep, and with marked success in certain cases.

In a very considerable number of cases, Dr. Lyons has found that a single dose of capsicum—twenty to thirty grains, according to the urgency of the symptoms—suffices to produce rest, sleep, and consciousness.

